# Efficacy of Single-Dose Azithromycin for Ocular Chlamydial Infection: A Longitudinal Study

**DOI:** 10.4269/ajtmh.23-0540

**Published:** 2024-03-19

**Authors:** Neha Pondicherry, Amza Abdou, Boubacar Kadri, Beido Nassirou, Sun Y. Cotter, Nicole E. Varnado, Travis C. Porco, Sheila K. West, Thomas M. Lietman, Jeremy D. Keenan

**Affiliations:** ^1^ Francis I. Proctor Foundation, University of California, San Francisco, California;; ^2^ Programme National de Santé Oculaire, Niamey, Niger;; ^3^ Department of Ophthalmology, University of California, San Francisco, California;; ^4^ Dana Center for Preventive Ophthalmology, Wilmer Eye Institute, Johns Hopkins University, Baltimore, Maryland;; ^5^ Department of Epidemiology & Biostatistics, University of California, San Francisco, California;; ^6^ Institute for Global Health, University of California, San Francisco, California

## Abstract

Millions of doses of azithromycin are distributed each year for trachoma, yet the treatment efficacy of a single dose of azithromycin for ocular *Chlamydia* infection has not been well characterized. In this study, four villages in Niger received a mass azithromycin distribution for trachoma. All 426 children aged 0–5 years residing in the study villages were offered conjunctival swabbing every 6 months to test for ocular *Chlamydia trachomatis*. Among the children infected with ocular *Chlamydia* before treatment, 6% (95% CI: 2–15%) tested positive for ocular *Chlamydia* infection 6 months later, and 15% (95% CI: 7–28%) tested positive 12 months later. The most important predictor of post-treatment ocular *Chlamydia* infection was pretreatment ocular *Chlamydia* infection (relative risk: 3.5, 95% CI: 1.3–9.4). Although the 6-monthly monitoring schedule was suboptimal for testing the treatment efficacy of an antibiotic, these findings are nonetheless consistent with high treatment efficacy of a single dose of azithromycin and suggest that additional interventions might be most effective if targeted to those children infected prior to treatment.

## INTRODUCTION

Trachoma, the leading infectious cause of blindness worldwide, is caused by ocular infection with *Chlamydia trachomatis*. As of July 2023, around 116 million people worldwide remained at risk.^1^ The WHO recommends 3–5 years of annual mass azithromycin distributions in districts where ≥10% of children aged 1–9 years have trachomatous inflammation–follicular (TF), according to the WHO’s simplified grading system.^2^ Despite the millions of doses of azithromycin distributed each year for trachoma, few studies have assessed the treatment efficacy of a single dose of azithromycin for an individual child with ocular *Chlamydia* infection.^3^^–^^5^ The objectives of the present study were to estimate the treatment efficacy of a single dose of azithromycin and to determine factors associated with an increased incidence of ocular *Chlamydia* infection.

## MATERIALS AND METHODS

**Figure 1.**
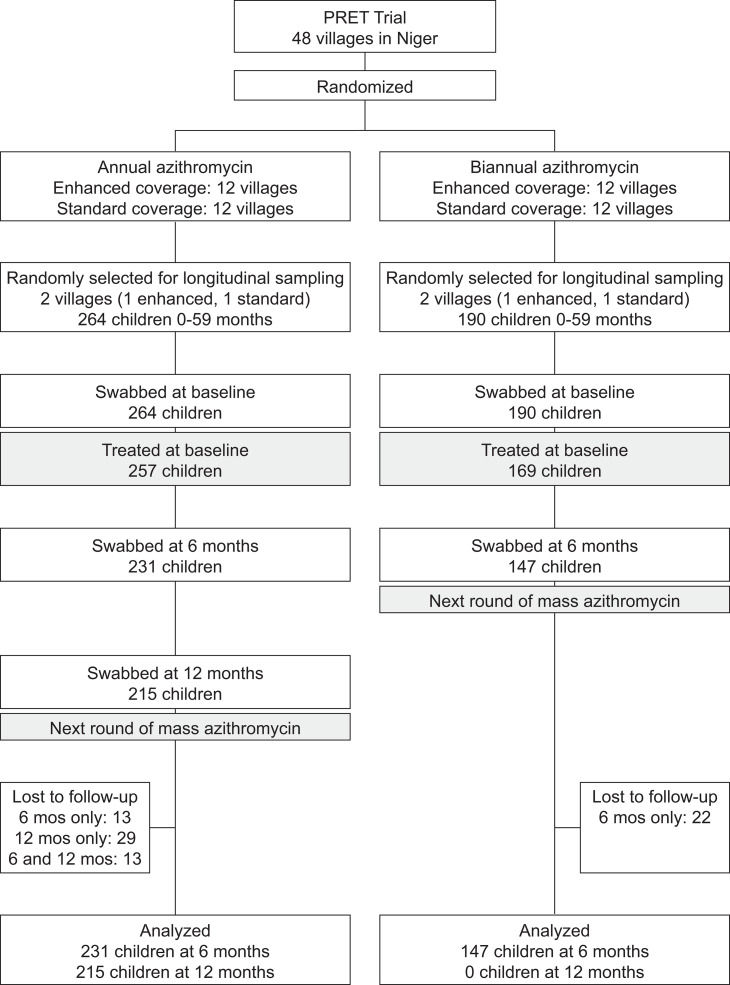
Study flow. PRET = Partnership for the Rapid Elimination of Trachoma trial.

This study describes a non-prespecified analysis of children treated with azithromycin in 2010–2011 at the Niger site of the cluster-randomized Partnership for the Rapid Elimination of Trachoma (PRET) trial.^6^ As part of the PRET trial, all residents from 48 villages in Matameye District, Zinder Region, Niger were enumerated on a door-to-door census. In 2010 the prevalence of TF among 0–5-year-olds in the study area was approximately 25%.^6^ Villages were subsequently randomized in a factorial design to annual versus biannual mass azithromycin distributions and to standard (i.e., ≥80%) versus enhanced (i.e., ≥90%) antibiotic coverage, with coverage targets based on the most recent study census. During the antibiotic distributions, a single dose of azithromycin was offered to each community member (1 g for adults, 20 mg/kg for children). Trachoma monitoring was performed at biannual study visits, with a new random sample of 0–5-year-old children selected from each village at each study visit. In addition, one village from each of the four treatment groups was randomly selected for longitudinal monitoring, in which the same children were followed up at each visit (Figure 1). Here, we report results from these four longitudinally monitored villages for the period after their first mass azithromycin distribution (i.e., months 0–12 for the two annually treated villages and months 0–6 for the two biannually treated villages).

At all monitoring visits, the upper right eyelid was everted and graded for the presence of TF and trachomatous inflammation–intense (TI) according to the WHO’s simplified grading system. Conjunctival swabs of the everted right superior tarsal conjunctiva were collected without media and transported to the University of California, San Francisco, where they were stored at −80°C. Swabs were pooled in groups of five and processed for *C. trachomatis* with the AMPLICOR polymerase chain reaction assay (Roche Diagnostics, Indianapolis, IN); swabs from positive pools were subsequently tested individually. Details of swab storage, transport, and processing are reported elsewhere.^6^ Log-binomial regression models incorporating a random intercept for a village were used to estimate incidences and to assess for risk factors of post-treatment infection at month 6 and month 12. Analyses were performed using R, version 4.1.3 (R Foundation for Statistical Computing) with a significance level of 0.05.

Ethical approval was obtained from the Committee on Human Research at the University of California, San Francisco and the Comité d’Ethique du Niger. Owing to the high prevalence of illiteracy in the study area, the ethical boards approved verbal consent, which was obtained from community leaders before randomization and from participants or a caregiver at the time of antibiotic distribution and examinations.

## RESULTS

At the baseline visit, 426 children aged 0–59 months from the four longitudinally monitored villages contributed a conjunctival swab and then received azithromycin treatment, of whom 378 (89%) were again swabbed 6 months later. Baseline characteristics were similar between children lost to follow-up and those remaining in follow-up (Table 1). Of the 378 children with a month 6 swab, 68 had evidence of *Chlamydia* infection at baseline, of whom four were infected 6 months after receiving azithromycin (6%, 95% CI: 2–15%). Conversely, of 310 uninfected children who received the baseline azithromycin treatment and participated in the month 6 monitoring, 11 were infected at month 6 (3%, 95% CI: 1–8%). The two biannually treated villages subsequently received a mass azithromycin distribution at month 6 and thus did not contribute data from the month 12 study visit for this analysis. In the two annually treated communities that did not receive additional azithromycin over the first year, 215 of the 257 (84%) children swabbed at baseline were again swabbed at month 12. Among these 215 children with 12-month follow-up, 48 were infected at baseline, of whom 7 also tested positive for *Chlamydia* at month 12 (15%, 95% CI: 7–28%), and 167 were not infected at baseline, of whom 7 were infected at month 12 (4%, 95% CI: 2–10%).

**Table 1:** Baseline characteristics of children who participated in follow-up visits and of those lost to follow-up at the 6-month and 12-month study visits

Pretreatment Exposure	6 Months after Treatment in Four Villages		12 Months after Treatment in Two Villages	
	Participated (*n* = 378)	LTFU (*n* = 48)	Participated (*n* = 215)	LTFU (*n* = 42)
Age				
<1 year	49 (13%)	9 (19%)	25 (12%)	5 (12%)
1 year	69 (18%)	11 (23%)	46 (21%)	3 (7%)
2 years	79 (21%)	12 (25%)	50 (23%)	15 (36%)
3 years	97 (26%)	8 (17%)	52 (24%)	10 (24%)
4 years	84 (22%)	8 (17%)	42 (20%)	9 (21%)
Sex				
Female	186 (49%)	21 (44%)	109 (51%)	16 (38%)
Male	192 (51%)	27 (56%)	106 (49%)	26 (62%)
TF				
Absent	274 (72%)	43 (90%)	149 (69%)	29 (69%)
Present	104 (28%)	5 (10%)	66 (31%)	13 (31%)
TI				
Absent	340 (90%)	45 (94%)	192 (89%)	39 (93%)
Present	38 (10%)	3 (6%)	23 (11%)	3 (7%)
CT				
Negative	310 (82%)	41 (85%)	167 (78%)	30 (71%)
Positive	68 (18%)	7 (15%)	48 (22%)	12 (29%)
Sibling with CT				
Yes	237 (63%)	31 (65%)	117 (54%)	26 (62%)
No	141 (37%)	17 (35%)	98 (46%)	16 (38%)

The values in the table are numbers (proportion). CT = ocular *Chlamydia trachomatis*; LTFU = lost to follow-up; TF = trachomatous inflammation–follicular; TI = trachomatous inflammation–intense.

Associations between pretreatment exposures and ocular *Chlamydia* outcomes 6 and 12 months after treatment are shown in Table 2. The most important predictor of post-treatment ocular *Chlamydia* infection was pretreatment ocular *Chlamydia* infection, which conveyed a relative risk of 1.6 (95% CI: 0.5–4.7; *P* = 0.43) at 6 months and 3.5 (95% CI: 1.3–9.4; *P* = 0.01) at 12 months. None of the other potential exposure variables (e.g., sex, age, presence of TF at baseline, presence of TI at baseline, or presence of an infected sibling at baseline) had significant associations with post-treatment ocular *Chlamydia*.

**Table 2:** Associations between pretreatment exposures and ocular *Chlamydia* infection at 6 and 12 months after a single dose of azithromycin

Pretreatment Exposure	6 Months after Treatment, *n* = 4 Communities (*N* = 378 children)		12 Months after Treatment, *n* = 2 Communities (*N* = 215 children)	
	CT+/Total	PR (95% CI)	CT+/Total	PR (95% CI)
Age				
<1 year	3/49 (6%)	Reference	3/25 (12%)	Reference
1 year	0/69 (0%)	0.1 (0–0.1.0)*	1/46 (2%)	0.2 (0–1.7)
2 years	6/79 (8%)	1.2 (0.3–5.1)*	3/50 (6%)	0.5 (0.1–2.3)
3 years	4/97 (4%)	0.6 (0.15–3.0)*	4/52 (8%)	0.6 (0.2–2.7)
4 years	2/84 (2%)	0.4 (0.1–2.2)*	3/42 (7%)	0.6 (0.1–2.7)
Sex				
Female	11/186 (6%)	Reference	10/109 (9%)	Reference
Male	4/192 (2%)	0.3 (0.1–1.0)	4/106 (4%)	0.4 (0.1–1.3)
TF				
Absent	10/274 (4%)	Reference	9/149 (6%)	Reference
Present	5/104 (5%)	1.4 (0.5–3.8)	5/66 (8%)	1.3 (0.4–3.6)
TI				
Absent	13/340 (5%)	Reference	11/192 (6%)	Reference
Present	2/38 (4%)	1.7 (0.4–7.3)	3/23 (13%)	2.3 (0.7–7.6)
CT				
Negative	11/310 (4%)	Reference	7/167 (4%)	Reference
Positive	4/68 (6%)	1.6 (0.5–4.7)	7/48 (15%)	**3.5 (1.3–9.4)**
Sibling with CT				
Yes	6/237 (3%)	Reference	7/117 (6%)	Reference
No	9/141 (6%)	2.2 (0.8–6.3)	7/98 (7%)	1.2 (0.4–3.3)

CT = ocular *Chlamydia trachomatis*; PR = prevalence ratio from log-binomial regression model; TF = trachomatous inflammation–follicular; TI = trachomatous inflammation–intense. Boldface statistics = associations with *P* <0.05.

* Odds ratio estimated with Firth’s penalized logistic regression because of complete separation.

## DISCUSSION

The treatment efficacy of azithromycin for *C. trachomatis* has mostly been studied in the context of sexually transmitted infections. For sexually transmitted *Chlamydia*, a single dose of azithromycin is thought to be 90–95% effective for urogenital infections but only 75% effective for rectal *Chlamydia*.^7^^–^^9^ The limited studies that have assessed the treatment efficacy of azithromycin for ocular *Chlamydia* have had variable results, with testing done 2–6 months after drug administration, suggesting a treatment efficacy between 70% and 94% for single-dose therapy.^3^^,^^4^ The present study, conducted in a trachoma-mesoendemic setting during an initial round of mass antibiotic treatments, found that 94% of infected children tested negative for *Chlamydia* 6 months after a single dose of azithromycin and that the most important risk factor for being infected with ocular *Chlamydia* after a mass antibiotic distribution was ocular *Chlamydia* infection prior to treatment.

Post-treatment *Chlamydia* infection was more common among younger children and those with TF or TI at baseline, although the association with these baseline characteristics did not achieve statistical significance in the present study. The most important risk factor for post-treatment chlamydial infection in this population was the presence of ocular *Chlamydia* infection prior to treatment, a finding consistent with several previous studies.^10^^,^^11^ Thus, this study provides some evidence that additional interventions may be most effective if targeted to children with chlamydial infection prior to mass drug administration. Randomized trials are starting to test this hypothesis. In one trial set in a hyper-endemic area of Ethiopia, triannual azithromycin distributions were targeted only to those 0–5 years old with *Chlamydia* infection prior to a round of mass azithromycin treatment. Although this strategy was not found to be effective, the prevalence of ocular *Chlamydia* among older children not eligible for targeted treatments was high and likely led to increased transmission.^12^ Targeted treatments may be more effective if given as a supplement to routine annual mass azithromycin distributions. An ongoing trial is testing such a strategy (clinicaltrials.gov NCT03335072).

This study has limitations. Treatment efficacy was estimated based on ocular *Chlamydia* monitoring performed 6 and 12 months after treatment. The infrequency of visits may have increased the chances of misclassification as some children may have cleared their infection after receiving antibiotics but then been reinfected in the intervening 6 months, and others may have failed to clear their infection after treatment but nonetheless had spontaneous resolution of infection. However, prior longitudinal studies have suggested that the median duration of an ocular chlamydial infection is approximately 17 weeks, increasing the chances that a negative post-treatment test represents treatment success as well as the plausibility that an infection observed 6 months after treatment represents treatment failure.^13^ Genotypic results might have been useful to differentiate treatment failures from reinfections, and antimicrobial susceptibility results could have been useful to determine the likelihood of treatment failure, but neither was performed as part of this study. Although baseline characteristics were similar, it is possible that rates of reinfection could have been different for the approximately 10–15% of children lost to follow-up. Polymerase chain reaction tests, although highly sensitive and specific, are not perfect. The results are representative of the larger sample of villages enrolled in PRET and likely generalizable to other trachoma-endemic areas of Niger but may not be generalizable to areas with a different endemicity of trachoma or different patterns of trachoma transmission.^6^

## CONCLUSION

This study found that 94% of children infected at baseline no longer were infected with ocular *Chlamydia* 6 months after a single dose of azithromycin. The most important risk factor for ocular *Chlamydia* infection after a mass azithromycin treatment was ocular *Chlamydia* infection prior to treatment. If additional measures beyond annual mass antibiotic treatments are instituted to further interrupt *Chlamydia* transmission (e.g., supplemental antibiotic treatments), one approach might be to focus on those most likely to have pretreatment ocular *Chlamydia* infection, such as preschool children.
